# Recent Advances in Polymer Nanomaterials for Drug Delivery of Adjuvants in Colorectal Cancer Treatment: A Scientific-Technological Analysis and Review

**DOI:** 10.3390/molecules25102270

**Published:** 2020-05-12

**Authors:** Marlon Osorio, Estefanía Martinez, Tonny Naranjo, Cristina Castro

**Affiliations:** 1 School of Engineering, Universidad Pontificia Bolivariana, Circular 1 # 70-01, Medellín 050031, Colombia; marlonandres.osorio@upb.edu.co (M.O.); estefania.martinezc@upb.edu.co (E.M.); 2 School of Health Sciences, Universidad Pontificia Bolivariana, Calle 78 B # 72 A-109, Medellín 050034, Colombia; tonny.naranjo@upb.edu.co; 3 Medical and Experimental Mycology Group, Corporación para Investigaciones Biológicas, Carrera 72 A # 78 B-141, Medellín 050034, Colombia

**Keywords:** colorectal cancer, antioxidants, 5-fluorouracil, polymer nanomaterials, nanocapsules, chemotherapy

## Abstract

Colorectal cancer (CRC) is the type with the second highest morbidity. Recently, a great number of bioactive compounds and encapsulation techniques have been developed. Thus, this paper aims to review the drug delivery strategies for chemotherapy adjuvant treatments for CRC, including an initial scientific-technological analysis of the papers and patents related to cancer, CRC, and adjuvant treatments. For 2018, a total of 167,366 cancer-related papers and 306,240 patents were found. Adjuvant treatments represented 39.3% of the total CRC patents, indicating the importance of adjuvants in the prognosis of patients. Chemotherapy adjuvants can be divided into two groups, natural and synthetic (5-fluorouracil and derivatives). Both groups can be encapsulated using polymers. Polymer-based drug delivery systems can be classified according to polymer nature. From those, anionic polymers have garnered the most attention, because they are pH responsive. The use of polymers tailors the desorption profile, improving drug bioavailability and enhancing the local treatment of CRC via oral administration. Finally, it can be concluded that antioxidants are emerging compounds that can complement today’s chemotherapy treatments. In the long term, encapsulated antioxidants will replace synthetic drugs and will play an important role in curing CRC.

## 1. Introduction

Every sixth death in the world is due to cancer, making it the second leading cause of death [1,2], and despite survival rates increasing, according to the Institute for Health Metrics and Evaluation of the University of Seattle, more than a million people died because of cancer globally in 2017 [3]. In 2016, more than 8.15 million people suffered from breast cancer, which was the type with the most morbidity, followed by colorectal cancer (CRC) and prostate cancer with 6.32 million and 5.7 million cases, respectively [1]. Of these three cancer types, CRC represents the highest number of deaths [3]. Several factors increase the risk of cancer; however, advancing age is the most important for cancer overall, and for CRC, specifically, a diet rich in meat cooked at high temperatures is associated with an increased risk of CRC [4].

Moreover, moderate-to-heavy alcohol consumption is associated with a 1.2- to 1.5-fold increased risk of CRC [5]. Others factors include African American ethnicity, male sex, inflammatory bowel disease, obesity, a sedentary lifestyle, red meat and processed meat intake, tobacco use, a history of abdominal radiation, acromegaly, renal transplant with use of immunosuppressive medications, diabetes mellitus and insulin resistance, androgen deprivation therapy, cholecystectomy, coronary artery disease, and ureterocolic anastomosis [6].

All of the above have led to a huge interest in cancer research. Today, cancer research is focused on identifying the causes and developing strategies for the prevention, diagnosis, treatment, and cure of cancer [7]. Cancer is one of the most investigated subjects; for instance, the proportion of cancer-related entries in PubMed rose from 6% in 1950 to 16% in 2016 [8], being even higher than for other diseases such as various infections (malaria, AIDS, tuberculosis, among others) and diabetes [8].

Accordingly, there is huge scientific research interest in treating and curing CRC, and several strategies have arisen to improve the therapeutic effects and reduce the side effects of actual chemotherapy for CRC treatment. Most of them include polymer drug delivery systems. Thus, this paper aims to review polymer drug delivery systems for adjuvant treatments for CRC, including an initial scientific-technological analysis of the papers and patents related to cancer, CRC, and adjuvant treatments using polymer approaches. The novelty of this paper lies in its broad overview of polymer families and the interaction of them with adjuvants. Moreover, it is explained how the behavior of those emerging drug delivery strategies is related to the superficial charge and chemical groups of the used polymers, information that, to the knowledge of the authors, is not reviewed in the literature for CRC.

## 2. Scientific-Technological Analysis

**Figure 1:**
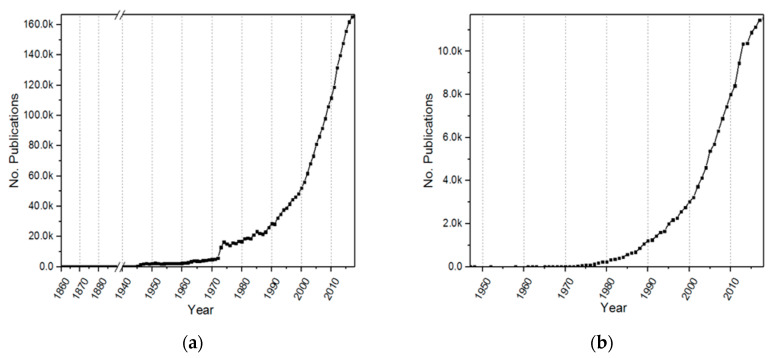
Number of publications of cancer in Scopus; (**a**) total number of cancer-related publications from 1860 to 2018 using the search string “TITLE-ABS-KEY (cancer)”; (**b**) total number of colorectal cancer-related publications from 1947 to 2018 using the search string “TITLE-ABS-KEY (colorectal and cancer)”.

In Scopus, the total of cancer-related papers in 2018 was 2,857,590 (see Figure 1a), and CRC-related papers numbered 167,366 (see Figure 1b); both subjects presented an exponential rise from 1970. The first documents indexed in Scopus related to CRC were report cases of medical interventions; for instance, Mr. Luke (1860) reported the surgery of an intestinal obstruction resulting from CRC [9]. Similarly, Dunphy (1947) reported four cases of CRC and included a gross pathological evaluation of the tumors [10].

In 1970, the first paper that reported the use of adjuvants was published by Adams et al. (1970), in which they compared the use of intralymphatic 5-fluorouracil (5-FU) and radioactive gold as adjuvants to surgical operations for colorectal carcinoma [11]. The next year, another two papers were published, and today, more than 550 papers have been published on this matter per year (Scopus, search string “TITLE-ABS-KEY (colorectal and cancer and adjuvants)”), representing more than 7% (about 11,700 research papers) of the overall CRC research.

**Figure 2:**
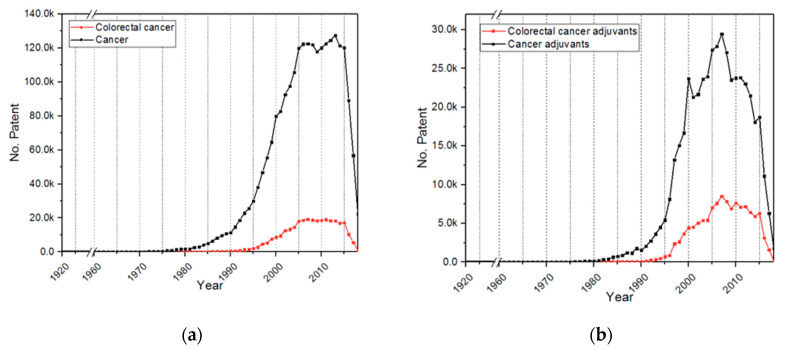
Number of patents per year in (**a**) cancer and colorectal cancer (CRC) (data found in AcclaimIP)—the words “cancer” and “colorectal cancer” were examined in the title, abstract and claims, separately; (**b**) cancer adjuvants and colorectal cancer adjuvants (data found in AcclaimIP)—the words “cancer adjuvant” and “colorectal cancer adjuvant” were searched for in the title, abstract and claims, separately.

Patents related to cancer represented around 2,356,397 entries from 1898 to 2018. The number of patents related to CRC is about 306,240. The pattern of the number of patents per year is presented in Figure 2. It was interesting to find that that the number of patents related to cancer is lower than the number of scientific papers; this behavior can be related to the number of documents of clinical study cases that count for the papers.

Regarding adjuvant therapies, it was found that adjuvants represent 20.0% of the overall cancer patents, and for CRC, adjuvants represent 39.3%. Thus, the number of patents related to adjuvants is proportionally higher for CRC (approximately two-fold) than for overall cancers, which can be explained by the motivation of scientists to cure the most deadly cancer type.

By comparing Figure 1 and Figure 2, it can be seen that adjuvants are more likely to be patented than to be scientifically published (12.6 K vs. 478.9 K) because scientists and companies prefer to protect their intellectual property that can be economically exploited. For instance, the global adjuvant market size was valued at USD 308.99 million in 2016 and is anticipated to grow with a compound annual growth rate (CAGR) of 10.6% [12]. Furthermore, it can be observed that while scientific papers grew rapidly, the number of patents grew until 2010 and then dropped. This behavior can be attributed to the recent difficulty of patenting because applicants’ claims should be new and clearly different to previous work [13], which is vast in cancer research, meaning that only 50% of patent applications are adjudicated. This is also discouraging new applications, since authors and companies do not see an attractive cost–benefit ratio for the patenting process [13].

**Figure 3:**
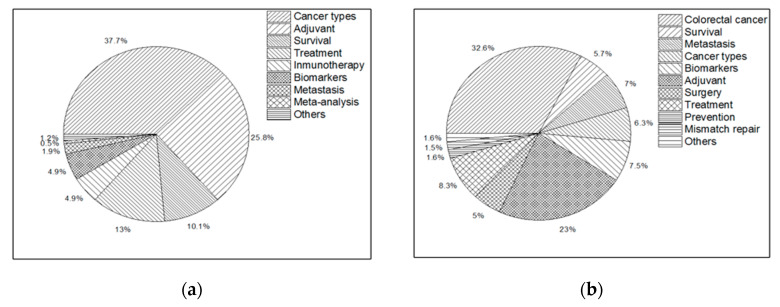

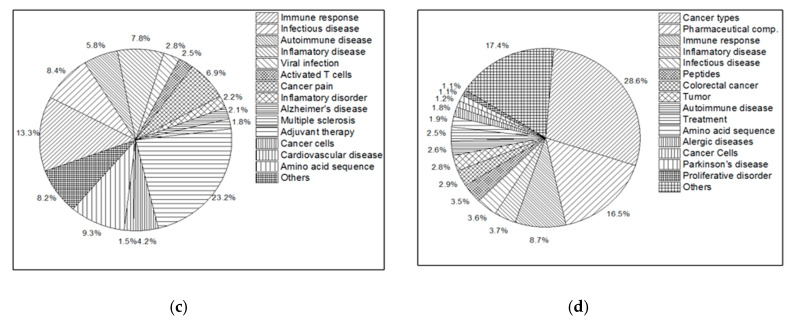
Keyword and title phrase groups in the latest papers and patents up to 31/12/2018; (**a**) grouped keywords of cancer adjuvants in scientific papers; (**b**) grouped keywords of colorectal cancer adjuvants in scientific papers; (**c**) grouped title phrases of cancer adjuvants in patents; (**d**) grouped title phrases of colorectal cancer adjuvants in patents. Authors’ keywords were extracted from 2000 scientific papers in Scopus and analyzed using VantagePoint. Title patent phrases were extracted from 1000 patents of Derwent and analyzed using VantagePoint.

By analyzing the keywords in the latest 2000 papers in Scopus and the title phrases in 1000 patents in the Derwent software (Clarivate Analytics, PA, USA) and then grouping the main subjects (see Figure 3), it was found that adjuvants are the second group for cancer and CRC, with 28.5% and 23.0% of entries, respectively, showing the importance of adjuvant therapy for improving the success of cancer treatments. Emerging strategies such as immunotherapy and biomarkers for treating and classifying cancer were also found.

Regarding the group of adjuvants, it was found that the terms “drug” and “chemotherapy” are more important for CRC than for cancer overall (see Table 1), again exposing the importance of drug delivery strategies for CRC. The drugs with more studies in recent papers (organized per decreasing number of entries) for CRC treatment are 5-fluoraucil, bevacizumab, capecitabine, metformin, aspirin, and irinotecan. Aspirin and metformin drew attention because they are drugs that are used for other purposes. For instance, aspirin and acetyl-salicylates, are used for treating fever, inflammation and pain [14]; however, Garcia et al. (2012) concluded that daily aspirin use at any dose was associated with a 21% lower risk of all-cancer death [15], and Chang et al. (2009) reported that aspirin reduces the risk of colorectal neoplasia in randomized trials and inhibits tumor growth and metastases in animal models, especially in tumors that overexpress cyclooxygenase-2, whereas aspirin can reduce that expression [16].

By analyzing the patents, it was found that the title phrases were more difficult to group and that the topics, in general, were more disperse (see Figure 3c,d); however, adjuvants and pharmaceutical compositions represent more than the 15% of the entries, coming in second place after cancer types, which includes the entries of all cancer names. Likewise, the drugs grouped in pharmaceutical composition for CRC are more diverse, but include indazoles derivatives, heteroaryl derivatives, heterocyclic compounds, quinoline compounds, and tumorigenic inhibitors, among others. These compounds are anticancer agents that have shown antiproliferative activity against cancer cells [17,18].

## 3. Cancer

According to the World Health Organization, “cancer is a generic term for a large group of diseases characterized by the growth of abnormal cells beyond their usual boundaries that can then invade adjoining parts of the body and/or spread to other organs” [19]. Cancer involves carcinogenesis, which means cancer development; more accuracy, carcinogenesis was first defined by Hecker in 1976 as the “generation of neoplasia” [20]. Neoplasia is an abnormal growth not coordinated with the surrounding tissue [21].

Cancer is initiated via carcinogens in the environment that induce mutations in critical genes, and these mutations direct the cell in which they occur, as well as all of its progeny cells, to grow abnormally. The result of this abnormal growth appears years later as a tumor [21].

**Figure 4:**
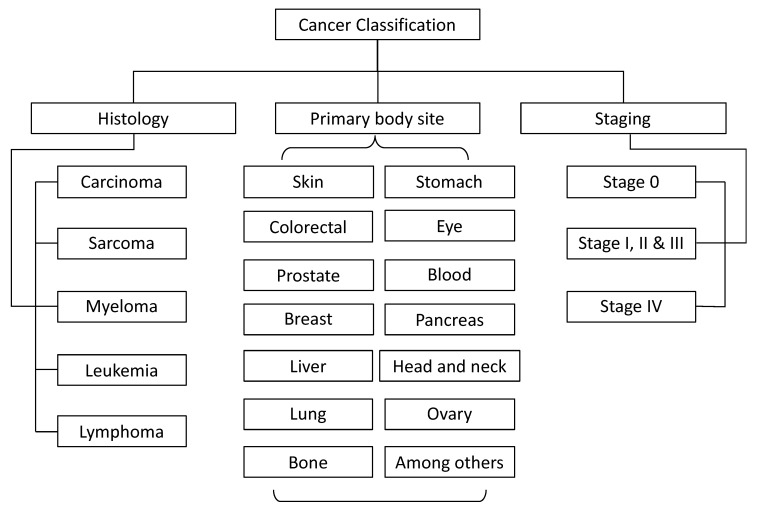
Traditionally cancer classification, histology, primary body site and staging.

Cancer has been traditionally classified in three ways: by the type of tissue in which cancer originates (histological type), by the primary body site, and by the staging (see Figure 4) [19,21].

Histologically, cancer is classified as carcinoma, sarcoma, myeloma, leukemia, and lymphoma [22,23]. Carcinoma refers to a malignant neoplasm of epithelial origin, skin, and tissues that line or cover internal organs. There are two subtypes, adenocarcinoma, which develops in an organ or gland, and squamous cell carcinoma, which originates in the squamous epithelium [23].

Sarcoma refers to cancer that originates in supportive and connective tissues such as the bones, tendons, cartilage, muscle, and fat [22]. Myeloma is cancer that originates in the plasma cells of the bone marrow. Leukemias are also presented in the bone marrow, but this cancer type is associated with the overproduction of immature white blood cells. Leukemia also affects red blood cells and can cause poor blood clotting and fatigue due to anemia [23].

Lymphomas develop in the glands or nodes of the lymphatic system—a network of vessels, nodes, and organs (specifically the spleen, tonsils, and thymus) that purify bodily fluids and produce infection-fighting white blood cells, or lymphocytes [22,23].

The body site classification is more familiar to patients, and it refers to the anatomical site at which the cancer appears, for instance, the brain, colorectal tissues, skin, or breast, among others. Finally, staging refers to the process of determining the size of cancer in the body and its localization [23]. To assess the staging of cancer, several diagnostic tests are required (X-rays, biopsies, and ultrasound, among others). In Stage 0, abnormal cells are present but have not spread to nearby tissue [24]; in Stages I, II, and III, cancer is present (the higher stage number, the larger the tumor and the more it has spread into surrounding tissues); and in stage IV, cancer has spread to distant parts of the body (metastasis) [23].

There are also other systems more precise from the medical point of view, for instance, the tumor-node-metastasis (TNM) system that is suggested by The American Joint Committee on Cancer. The TNM is updated periodically, based on advances in the understanding of cancer prognosis, to remain current and relevant for clinical practice; this manual is also described for every anatomical site [23,25].

Recently, there have been other efforts to generate an integrated system to classify cancer [26]. Cancer cells are classified using the cell morphology, leaving behind the functional attributes of these cancer cells. Using techniques in biology based on genomics, transcriptomics, and proteomics, scientists can model the attributes of cancer stem cells and their potential contribution to treatment responses and metastases [26,27,28,29,30].

## 4. Colorectal Cancer

The name “colorectal cancer” is used to describe bowel cancer that starts in the colon or the rectum. CRC almost always develops from growths called colorectal polyps that form in the lining of the colon (adenocarcinoma in more than 95% of cases) [22,31]. After the diagnosis, the treatment depends on various factors, including the stage of cancer. In the early stages of CRC, the tumor just needs to be surgically removed, but in advanced stages, additional treatments may be considered, such as chemotherapy and radiation therapy, among other adjuvant therapies [31].

## 5. Colorectal Cancer Treatments

As mentioned above, CRC treatment depends on cancer staging, but it is classified into two groups, surgery and adjuvant therapy.

### 5.1. Surgery

Colorectal cancers may be cured by surgery (hemicolectomy) [2], but only if the entire tumor is localized and no cells have spread to other sites (adenocarcinoma in situ) [22,32]. Moreover, in most of the cases, complementary therapies (adjuvants) are needed before or after surgery and vary from traditional to novel. Traditional chemotherapies are based on 5-FU, which inhibits cancer cell division. Nobody doubts that traditional adjuvant treatments have improved the prognosis of patients; however, they are not beneficial for all cases. For instance, not all women with breast cancer derive benefit from 5-FU-based adjuvant chemotherapy. Many older women (age > 70) with hormone receptor-positive early stage breast cancer treated with adjuvant chemotherapy will not accrue a survival advantage [33]. For CRC, it was demonstrated that extra cycles of traditional chemotherapy, either before or after chemoradiotherapy, have been shown not to improve survival compared with chemoradiotherapy alone. An argument for less chemotherapy is also presented by results from trials investigating neoadjuvant or preoperative therapy in esophageal and gastric cancers. In randomized trials, which enrolled patients with esophageal and gastro-esophageal junction adenocarcinoma, no survival benefit with four cycles of the ECX regimen (epirubicin, cisplatin, and capecitabine) were reported beyond that achieved with two cycles of fluorouracil and cisplatin. These results support the use of a shorter duration of preoperative chemotherapy [34].

5-FU can cause local and systematic toxicity (DNA damage) to healthy cells, generating undesired side effects, and favor the mutation of them to malignant cells. These problems can be overcome by using novel approaches such as targeting specific cancer-relevant proteins (such as oncogenic tyrosine kinases), immunotherapy, and reactive oxygen species (ROS)-modulated therapies, which are more specific in generating cancer cell apoptosis and reducing the side effects associated with traditional chemotherapy [35,36].

### 5.2. Adjuvant Therapy

Adjuvant therapy is defined by the United States National Cancer Institute (NCI) as “additional cancer treatment given after the primary treatment (surgery) to lower the risk that cancer will come back” [2], eliminating any residual microscopic cancer cells. Neoadjuvant therapy is an adjuvant therapy given before the primary treatment to make it more effective or easier [37]. Adjuvant therapies for CRC can be divided into five groups, i.e., radiation, hormones, targeted therapy, immunotherapy, and chemotherapy.

#### 5.2.1. Radiation Therapy

Radiation therapy (RT) is the application of ionizing radiation to treat cancer. Cancer cells are more sensitive to DNA damage than normal tissue cells; this characteristic provides the therapeutic effect [38]. It is estimated than 50% of the population with cancer can benefit from this therapy [38]. For colorectal cancer, RT is used as a neoadjuvant treatment to improve patient prognosis, heightening the overall survival rate, diminishing the local recurrence rate, and improving the quality of the surgical procedures [39].

Besides some side effects—such as incontinence of the anal sphincter or urinary tract problems, vaginal dryness, and sexual dysfunction, among others—related to neoadjuvant RT, new techniques have focused efforts on modulating the beam intensity and improving the beam precision and development of delivery systems for encapsulated radionuclides (brachytherapy) [39,40].

#### 5.2.2. Hormone Therapy

Bowel health largely depend on hormones; for instance, Jhonson et al. (2009) found that sex hormones such as estrogen and progestin are related to protective effects against CRC in menopausal women [41]. Similar results were found by Rennert et al. (2009) in 5214 women in perimenopausal/postmenopausal stages with CRC, where the use of estrogen/progestin replacement therapy reduced the risk of CRC by 63% [41]. For men, Lin et al. (2014) found that higher levels of testosterone are related to lower risks of CRC [42]. Hormone therapy is a systemic approach with several side effects such as abdominal pain, headache, depression, acne, nausea, leg cramps, and stroke, among others [43,44]. The use of drug delivery systems can help in reducing the needed doses, which can lower the undesired side effects of the treatment [45].

#### 5.2.3. Targeted Therapy

Targeted therapy is a type of treatment that uses drugs or other substances to identify and attack specific types of cancer cell with less harm to healthy cells. Targeted therapy may have fewer side effects than other types of cancer treatment. Targeted therapies are either small molecule drugs or monoclonal antibodies [23]. The most investigated monoclonal antibodies are bevacizumab, cetuximab, and panitumumab [46,47].

Bevacizumab is a recombinant humanized monoclonal IgG antibody that selectively binds to vascular endothelial growth factor A (VEGF-A), and it demonstrates anti-tumor activity by blocking vascular endothelial growth factor receptor 2 (VEGFR2). Furthermore, patients treated with cetuximab and panitumumab showed a survival benefit in metastatic CRC [46]. Cetuximab is an anti-epithelial grow factor (EGFR) monoclonal antibody of the IgG1 class targeted against the extracellular domain of the EGFR. By binding to the EGFR, cetuximab blocks intracellular EGFR signaling and modulates tumor cell growth by inhibiting proliferation, angiogenesis, and differentiation; stimulating apoptosis; and preventing metastasis. Panitumumab is a fully human, monoclonal antibody targeting the EGFR with high affinity. Its mechanism of inhibiting the EGFR signaling pathway is similar to that of cetuximab, as described above [46]. Nevertheless, to improve the efficacy of the treatment, the therapy should be complemented with cytotoxic chemotherapy using 5-FU [48,49].

#### 5.2.4. Immunotherapy

Immunotherapy was first proposed in 1909 by Nobel Prize winner Paul Ehrlich. The idea behind it is that immune cells can control malignant cells and eradicate cancers before they manifest clinically [35]. However, in spite of several mechanisms active in the immune system to recognize and eliminate tumor cells, some variants of these cells selectively acquire increased resistance against immune responses. Thereafter, resistant cells continue to grow, evading the immune responses, and tumor cells develop resistance against both innate and adaptive immune mechanisms (cancer immunoediting) [35]. To avoid cancer immunoediting, patients can be vaccinated (monoclonal antibodies, adaptive T cells, DNA viral vectors, heat shock proteins, and dendritic cells, among others [35,50,51,52,53,54]) to raise specific immune responses. Moreover, this therapy is followed in parallel with another adjuvant therapy to potentiate the immune system [35].

#### 5.2.5. Chemotherapy

Chemotherapy for cancer treatment uses drugs (plant-derived or synthetic) called cytostatic drugs (cytotoxic chemotherapy), which aim to stop cancer cells from continuing to divide uncontrollably [55].

It is estimated that 20–30% of newly diagnosed patients with CRC present with unresectable metastatic disease. In addition, a considerable proportion of patients (40–50%) experience disease recurrence after surgical resection or develop metastatic disease, typically in the liver or lungs [56]. To improve the life prognosis of those patients, several drugs have been developed, such as 5-FU, which is considered the gold standard for CRC chemotherapy [56].

5-FU was developed in 1957 by Charles Heidelberger and colleagues at the University of Wisconsin, who observed that tumor tissues preferentially used uracil-type molecules for nucleic acid biosynthesis and postulated that a fluorouracil analog would be easily taken up by cancer cells. Likewise, it would inhibit tumor cell division by blocking the conversion of deoxyuridine monophosphate (dUMP) to deoxythymidine monophosphate (thymidylate) [56].

From 1957 to date, 5-FU has been complemented with other adjuvants to improve the overall survival of patients. For instance, Petrelli et al. (1987) found that mixing 5-FU with leucovorin at 500 mg/m^2^ in metastatic patients improved overall survival [57]. Goldberg et al. (2004) studied the efficacy in metastatic CRC patients of 5-FU plus leucovorin, irinotecan, and oxaliplatin combinations (FOLFOX) in 795 patients, finding a median survival rate of 19.5 months, which is 35% higher compared with that with other treatments [58], and recently, Magne et al. (2012) investigated the efficacy of cetuximab with continuous or intermittent 5-FU, leucovorin, and oxaliplatin (Nordic FLOX) treatment versus FLOX alone in the first-line treatment of metastatic CRC, finding an overall survival of up to 20.4 months [59].

Today, there is a growing interest in researching natural drugs as adjuvants for CRC; most of them act against reactive oxygen species (ROS). Reactive oxygen species (ROS) include oxygen molecules, superoxide anion radicals, hydroxyl free radicals, and hydrogen peroxide. ROS are generated in the mitochondrial respiratory pathway. Although an increase in the level of intracellular ROS leads to oxidative stress and DNA damage, the effects of ROS are normally balanced by antioxidants, such as reduced glutathione (GSH), ascorbic acid, and uric acid [60]. Disruption of the oxidant–antioxidant balance through alterations to cellular homeostasis or the defective repair of ROS-induced damage is involved in carcinogenesis. Furthermore, it is known that anticancer drugs induce oxidative stress in patients with cancer being treated with chemotherapy [60]. To reduce oxidative stress, investigations are focusing on natural antioxidants. In Table 2, the latest studies for CRC using natural antioxidants are presented. Natural antioxidants are presented as extracts (fruits, plants, and coffee, among others) or polyphenol fractions from those extracts [61].

Accordingly, most of the antioxidants in Table 2 are polyphenols, due to most plant-based food naturally containing them. The basic monomer in polyphenols is a phenolic ring, and generally, these are classified as phenolic acids and phenolic alcohols [61]. Polyphenol consumption is strongly associated with a low cancer risk. For instance, the Mediterranean diet (rich in olive oil polyphenols [91]), reduces the risk of CRC by approximately 4% [92]. However, 4% is still modest; thus, polyphenols are extracted to present higher antioxidant activity and consequentially higher anticancer effects. Moreover, the colorectal anticancer effect can be potentiated if the antioxidant is supplied using a drug delivery system [93].

## 6. Polymer-Based Drug Delivery Systems for Adjuvants for Colorectal Cancer

Ideally, drugs would target the cancer cells with the exact therapeutic concentration. However, drug delivery is not easily controlled. Drug release rates, cell- and tissue-specific targeting, and drug stability are difficult to predict [93]. Furthermore, when targeting colon cells, the drug may avoid degradation and/or be released early, which would reduce its therapeutic effect.

Likewise, natural and synthetic compounds can be easily degraded by air, UV light, and moisture, and lose their antioxidant potential [94]. Thus, encapsulation is important for improving their stability and, overall, generating long-term desorption profiles that improve the CRC adjuvant treatments.

### 6.1. Nanoencapsulation

Nanoencapsulation is a nanostructured drug delivery system (10–1000 nm [95]) that can be loaded with small molecules or macromolecules, thus acting as a vehicle for chemotherapeutic drugs. Such materials are able to transport chemotherapeutic molecules to the desired area, increasing the drug concentration, to be subsequently released in a controlled manner. A great number of nanoformulations—such as liposomes, micelles, nanoemulsions, and polymeric nanoparticles, among others—have been reported as drug delivery systems to be applied in cancer treatment [96,97,98,99].

**Figure 5:**
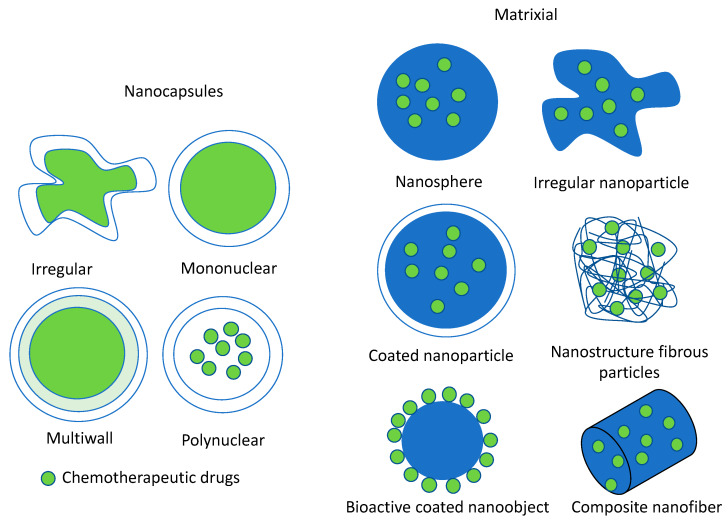
Nanodevices for the encapsulation of chemotherapeutic drugs/antioxidants.

Nanoencapsulation can be performed to generate two categories of nanodevice (see Figure 5), nanocapsules and matrixial nanomaterials. In the nanocapsules group, the chemotherapeutic drug is surrounded by a wall or shell material to generate spheres or irregular nanocapsules where the chemotherapeutic drug can be mononucleated (a single core) or polynucleated (multicore) [100,101,102].

Matrixial nanomaterials are more varied. Generally, the bioactive compound (chemotherapeutic drug) is embedded or superficially adsorbed in a polymer matrix. The polymer matrix can be configured in different forms, nanospheres, irregular nanoparticles, and nanofibers, among others [103,104,105]. Likewise, the nanoparticle may or not may be coated by another polymer. The nanoparticle can be solid or nanostructured by fibers [106,107].

The techniques used for achieving nanoencapsulation are complex. This is mainly due to the difficulty in attaining the complex morphology of the capsule and core material and the demands of controlling the release rate of the nanocapsules [108]. Various techniques have been developed and used for nanoencapsulation purposes. For instance, emulsification, coacervation, inclusion complexation, solvent evaporation, nanoprecipitation, and supercritical fluid techniques can produce capsules in the nanometer range (10–1000 nm) [95,108]. Most of the documents regarding encapsulation relate to particles between 100 and 1000 nm. However, there also some reports with capsules ranging from 10 to 100 nm.

### 6.2. Release Mechanism

**Figure 6:**
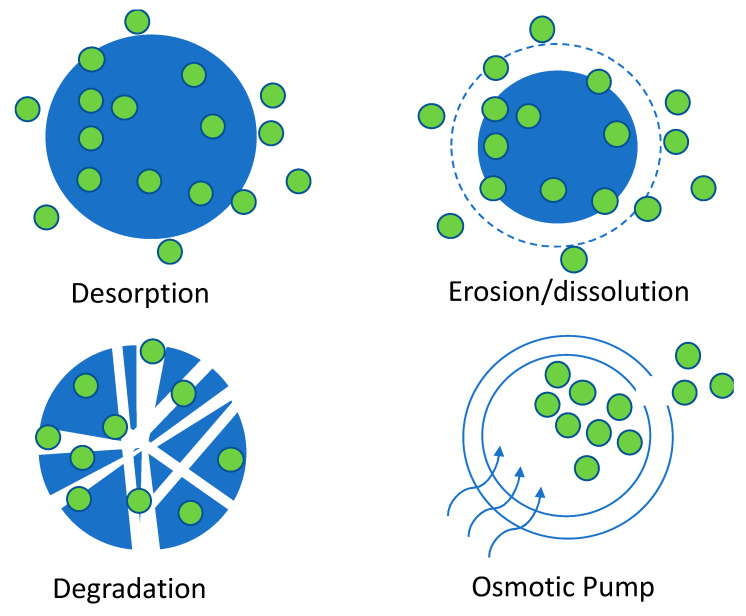
Drug-releasing mechanism of chemotherapeutic drugs.

Nanodevices (nanocapsules and matrixial nanomaterials) can provide several forms of release for the chemotherapeutic drugs (see Figure 6). Drugs can be desorbed from the matrix or core reservoir (nanocapsules) because of a concentration gradient that can be assisted by swelling or material relaxation, facilitating the release of the bioactive component [109].

The erosion/dissolution implies the loss of the shell or matrix integrity to favor the diffusion of the bioactive component. The erosion/dissolution of the nanodevice can be triggered by pH changes and water content, among others. Usually these systems deliver the bioactive compound quickly once the capsule is in contact with the target environment. Otherwise, the degradation mechanism tends to be a slow as it is mediated by enzymatic reactions [110,111].

An osmotically active drug can be delivered using an osmotic pump, in which the bioactive component is pushed away by a fluid that goes into the capsule via a semipermeable membrane and force the drug to pass throughout an orifice [112].

Along with the nanodevice type, the interaction of the biomaterial/bioactive compound modulates the releasing profile. Bioactive components for CRC treatment are complex; for instance, antioxidants poses aromatic rings with hydroxyl lateral groups, conferring to them a hydrophilic nature [113], and hydroxyl groups can easily generate hydrogen bond interactions with biomaterials. Antioxidants of high molecular weight present an amphiphilic nature, with hydrophilic and lipophilic zones such in the case of vitamin E and carotenoids [114], which can interact with either polar or nonpolar polymers.

Plant extracts contains several bioactive compounds (mixtures of hydrophilic and lipophilic compounds). Nevertheless, most CRC adjuvant extracts are, in general, hydrophilic in nature and water-soluble [90,115,116,117] and thus can interact with hydrophilic polymers. Likewise, synthetic bioactive compounds have an amphiphilic nature; for instance, 5-FU is a pyrimidine with oxygen and flour lateral groups, conferring it with a hydrophilic nature, but 5-FU can present resonance diminishing its water solubility [118]. According to the above, given the diversity of CRC adjuvants, several polymer-based biomaterials have been used for generating nanodevices for drug delivery systems.

### 6.3. Polymers for Oral Drug Delivery Systems

Biomaterials have improved the delivery and efficacy of a range of pharmaceutical compounds. In particular, polymer- and lipid-based materials have been designed to release therapeutics for extended periods of time and for targeting specific locations within the body, thereby reducing the toxicity to the patient whilst keeping the therapeutic effect [110]. Lipid-based drug delivery systems are beyond of the goals of this paper; these formulations types are reviewed in the following references [119,120,121]. For polymer drug delivery, the oral route has been proven to be most convenient route for chronic drug therapy [119]. For instance, in studies of CRC patients, it has been proven that the oral administration of adjuvant treatments is most suitable for the patient and cost-saving for health systems [122,123]. However, oral administration is challenging for CRC as the drug needs to be protected during passage through the digestive tract before proper delivery. For this application, polymers are advantageous due to their processability at the nanoscale, their wide range of functional groups, and the possibility of generating mixtures, composites, and copolymers, among others [124], favoring the protection of the drug and its delivery profile.

**Figure 7:**
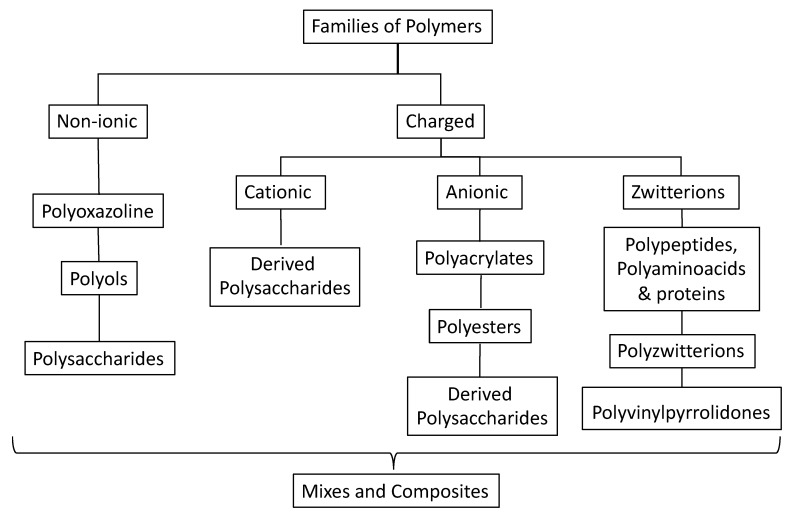
Polymer families for drug delivery systems of antioxidants for CRC.

Drug delivery polymers for colorectal cancer can be classified according to polymer nature, into non-charged polymers and charged polymers (anionic, cationic, and zwitterionic), as presented in Figure 7. Non-charged polymers cannot be charged via dissociation; thus, they are strongly stable at any pH value, and they can interact via hydrogen bonding and Van der Waals interactions. Alternatively, charged polymers can generate anionic, cationic, or zwitterionic charges on their surface and can switch from neutral to charged, depending on the hydrogen potential of the surrounding environment. The switching from neutral to charged will influence the chain polymer organization. For instance, Han et al. (2016) found that the carboxylic lateral groups of polyacrylic acid copolymers induces polymer changes in terms of roughness, thickness, and porosity, from pH 5.5 to 9 [125]. Those systems have the advantage of modulating the drug release by pH, as presented in the gastrointestinal fluids. The interaction with these polymers is more likely to be ionic, which is stronger, but requires at least polar or ionic charges in the bioactive compounds.

Mixtures and composites are alternative strategies for creating a synergistic response in the system [126,127]. In the following sections, the strategies for each polymer type in drug delivery systems for CRC are described.

### 6.4. Polymers for Encapsulating Antioxidants for Colorectal Cancer

Antioxidants for drug delivery have more than 4600 documents indexed in Scopus, with an accelerated growth from 2000 to date. From those, around 44.6% include the word “polymer”, which hints at the relevance of creating polymer-based drug delivery systems to protect these compounds. For encapsulating antioxidants, all polymer families have been used for the drug delivery of these compounds.

#### 6.4.1. Polysaccharides and Derived Polysaccharides

Polysaccharides are carbohydrate polymers composed of long chains of monosaccharides, such as glucose, fructose, and galactose, among others [128]. Polysaccharides can respond to pH, colon enzyme degradation, or peristaltic movement. For instance, chitosan can response to pH changes, starches can be degraded by amylase enzymes, and ethyl cellulose can be broken by colon waves [129]. Polysaccharides have garnered attention because, like antioxidants, they come from natural sources, allowing the development of bio-based therapies for CRC. Antioxidants have been successfully encapsulated using polysaccharides for nanocapsules such as cellulose, chitosan, and alginate, among others.

Cellulose is the most abundant polymer on earth; it is composed of β1-4 linked d (+) glucose [124,130,131]. Cellulose and its derivatives have been reported as carriers of antioxidants. For instance, Sunnasee et al. (2019) grafted β-cyclodextrins in cellulose nanocrystals, demonstrating that this system is not immunogenic and does not induce oxidative stress in the cell, thus it is safe for intracellular drug delivery [132]. Li et al. (2019) developed a nanoformulation of quercetin and cellulose nanofibers with sustained antioxidant activity. The nanocellulose fibers were an effective nanocarrier of the antioxidant with a loading capacity of 78.91% and encapsulation efficiency of 88.77%; moreover, the quercetin delivery profile was extended to higher times [133]. Ching et al. (2019) encapsulated curcumin in cellulose nanocrystals (via acid hydrolysis), adding surfactants to improve the loading capacity of the release profile of the antioxidant [134]. Ngwabebhoh et al. (2018) developed a Pickering suspension for encapsulating curcumin using cellulose nanocrystrals [106], finding that the capsules were stable for up to 8 days at different pHs [106]. However, the application was directed to antimicrobial properties instead of antioxidants for CRC.

Chitosan is a polysaccharide obtained from chitin deacetylation, composed of β-(1→4)-linked d-glucosamine (deacetylated unit) and *N*-acetyl-d-glucosamine (acetylated unit) [135]. It is a cationic polymer that responds to pH changes and can be converted into hydrogels, making this polymer attractive for the drug delivery of antioxidants. For instance, Kumar et al. (2015) encapsulated naringenin (polyphenol) using chitosan nanocapsules. Their studies proved that encapsulated naringenin has a better anticancer effect than free naringenin [136]. Jeong et al. (2016) crosslinked chitosan with resveratrol modified with phospholipids to improve its oral bioavailability and low water solubility. The researchers found an encapsulation efficiency of up to 85.59%, and an in vitro drug release study suggested a slow and a sustained release governed by diffusion [137].

Shi et al. (2017) encapsulated β-carotene and anthocyanin in (2,2,6,6-Tetramethylpiperidin-1-yl)oxyl (TEMPO)oxidized polysaccharides, specifically Konjac Glucomannan, in which some polysaccharides’ hydroxyl lateral groups were converted into carbonyl groups, and then the spheres were coated with chitosan. The advantage of the system was its ability to generate oil–water stable systems and to retain the antioxidants at gastric pH values; moreover, the capsules exhibited anticancer effects [138].

Alginates are salts derived from alginic acid, in which the polymer has a carbonyl lateral group liked to a glucose unit, making the polymer negatively charged [139]. Sookkasem et al. (2015) developed novel alginate beads for encapsulating curcumin for colon target therapy; the capsule was able to prevent release in the upper gastrointestinal tract and immediately release the drug upon the arrival of the beads in the colon [139]. However, the approach of Sookkasem et al. was not at the nanoscale; the alginate beads had a diameter in millimeters (macroscale). Similarly, Wang et al. (2019) developed a macroscale capsule but using ZnO instead of Ca^2+^ as the crosslinking agent to improve the release profile to a longer time [140]. Approaches at the nanoscale for this polymer have been tested using mixtures and composites, especially with chitosan [141,142], as reported in the following sections.

Maltodextrin (α 1–4 d (+) glucose) is a low molecular weight polysaccharide that has been broadly used for the encapsulation of food and pharmaceutical ingredients [143]. Ming et al. (2018) encapsulated red ginseng extract, generating a water/oil emulsion, and then coated it with maltodextrin using spray-drying. The researchers found particles ranging from 58 to 400 nm and optimized the conditions to produce them [143]; however, they did not evaluate the release or anticancer effects of the produced nanocapsules.

Pan et al. (2018) encapsulated curcumin in soybean polysaccharides (mixture of cellulose, xylogalacturonan, and arabinogalactan, among others), polysaccharides that easily link lipophilic compounds. The capsules were stable at a pH ranging from 2.0 to 7.0 [144]. Li et al. (2017) encapsulated phenolic acid antioxidants to prove the effectiveness of producing hollow Arabic gum and short linear glucans from starch templates. Hollow nanocapsules enhanced the antioxidant activity of the phenolic acids and improved the stability of their antioxidant activity in challenging environments with high salt concentrations, and when exposed to UV radiation and high temperatures [145].

Assis et al. (2017) incorporated lycopene nanocapsules in starch films to generate an edible film material, but the application was focused on packaging instead of biomedical efficacy [146]. Amah et al. (2019) encapsulated catechin in starch-based nanoparticles, providing protection to the catechin against the harsh gastric environment and helping to retain its bioactive properties during an in vitro digestion process [147]. Jana et al. (2017) reviewed different strategies for generating three-stimuli-responsive guar gum composites (pH, time, and enzymes) for colon-specific drug delivery [148]. Finally, Saffarzadeh-Matin et al. (2017) prepared an apple pomace polyphenolic extract and encapsulated it in maltodextrin in nanocapsules of 52 nm diameter, optimizing the loading efficiency of the process [149].

#### 6.4.2. Polyacrylates

Polyacrylates are polymers derived from acrylic acid via free radical polymerization. Nevertheless, Garay-Jimenez et al. (2011) reported an alternative method for producing polyacrylate nanoparticles between 40 and 50 nm by emulsion polymerization using a 7:3 mixture of butyl acrylate and styrene in water containing sodium dodecyl sulfate as a surfactant and potassium persulfate as a water-soluble radical initiator. The resulting method seemed promising for the encapsulation of bioactive agents of interest in the biomedical field [150].

Polyacrylates are anionic polymers that present maximum swelling at pH neutral to alkaline, and minimum under acidic conditions, making them ideal for colon-specific drug delivery systems [151].

Feuser et al. (2016) modified with folic acid polymethyl meta acrylate and ferrous sulfate to produce superparamagnetic nanoparticles with excellent colloidal stability and high efficacy in encapsulating lauryl gallate (antioxidant). The release profile of lauryl gallate showed an initial burst effect followed by a slow and sustained release, indicating a biphasic release system. The lauryl gallate loaded in superparamagnetic polymethyl methacrylate (PMMA) nanoparticles did not have any cytotoxic effects on non-tumoral cells. Moreover, the folic acid promoted folate receptor-mediated endocytosis in tumoral cells, enhancing the anticancer effect of lauryl gallate [152].

Ramalingam et al. (2018) loaded curcumin in electrospun fibers to generate a dressing for the treatment of skin cancer. The dressing induced cell proliferation and free radical scavenging activity and up-regulated the expression of CDKN2A in A375 melanoma cells. The cell death of A375 melanoma cells was dose- and time-dependent, which indicates that treatment with curcumin loaded in the nanofibers inhibited the growth and induced the cell death of the skin cancer cells [151].

Ballestri et al. (2018) developed a synthetic antioxidant (porphyrin) and encapsulated it in polymethyl methacrylate (PMMA) core-shell nanoparticle (70 nm diameter) for photodynamic cancer therapy. Photodynamic cancer therapy uses a light to activate an antioxidant compound. The capsule protected porphyrin against unwanted bleaching while preserving the anticancer activity, which was similar to that of free porphyrin under in vitro testing [153].

Recently, Sobh et al. (2019) synthesized a multi-walled carbon nanotube (MWCNT)/Poly(methyl methacrylate-co-2-hydroxyethyl methacrylate) P(MMA-co-HEMA) nanocomposite loaded with either curcumin or its water-soluble derivative via an in situ microemulsion polymerization technique by using different ratios of multi-walled carbon nanotube MWCNT to drug. Curcumin could be loaded in higher amounts with high entrapment efficiency values with improved thermal stability with an increased MWCNT ratio. The in vitro drug release studies of the nanocomposite showed a prolonged controlled release in the intestinal fluid at pH 7.4 and that ≤ 8% of the drug was lost in the stomach fluid at pH 1.2 [154].

Sunoqrot et al. (2019) developed a pH-sensitive polymeric nanoparticle of quercetin as a potential colon cancer-targeted nanomedicine. Quercetin is an abundant plant polyphenol with demonstrated efficacy in CRC. The researcher developed polymeric nanoparticles of quercetin based on the pH-sensitive polymer methacrylic acid and copolymers (Eudragit^®^ S100) to achieve colon pH-specific drug release. The researchers found nanoparticles with a mean diameter of 66.8 nm and a partially negative surface charge of −5.2 mV. In vitro release testing showed a delay in drug release in acidic pH but complete release within 24 h at pH 7.2. A cytotoxicity assay on CT26 murine colon carcinoma cells displayed a significantly higher potency of encapsulated quercetin (IC50 = 0.8 μM) than free quercetin (IC50 = 65.1 μM) [155].

#### 6.4.3. Polyols

Polyols are polymers with hydroxyl groups [156]. A representative polymer of this family is the polyvinyl alcohol (PVA), which is synthesized by the hydrolysis of polyvinyl acetate [157,158]. PVA is a water-soluble polymer that can generate hydrogels by chemical or physical crosslinking [159]. Recent advances are presented below.

Li et al. (2019) improved the solubility of curcumin using d-α-Tocopherol polyethylene glycol 1000 succinate (TPGS), a water-soluble derivative of vitamin E that acts as a surfactant with the ability to form micellar nanoparticles in water. More importantly, TPGS acts as a potent antioxidant. The complex TPGS/curcumin was encapsulated using PVA, in which were obtained stable nanoparticles of 12 nm diameter. The nanoparticle satisfactorily released curcumin in simulated colonic and gastric fluids; furthermore, the nanoparticles decreased intracellular ROS levels and apoptosis and inhibited the migration of HT-29 human colon cancer cells more potently than free curcumin. The pharmacokinetic analysis demonstrated that the nanocapsules were more bioavailable than free curcumin after oral administration to rats [160].

Wen et al. (2019) developed a core-shell electrospun nanofiber, core (PVA and phycocyanin), and shell (Polyoxyethylene) for the targeted therapy of CRC. Phycocyanin (PC), a water-soluble biliprotein (antioxidant), exhibits potent anti-colon cancer properties. The PC-loaded electrospun fiber mat inhibited HCT116 cell growth in a dose-dependent and time-dependent manner. In particular, the PC-loaded mat exerted its anticancer activity by blocking the cell cycle at the G0/G1 phase and inducing cell apoptosis, involving a decrease in Bcl-2/Bax, the activation of caspase 3, and the release of cytochrome c [161].

Golkar et al. (2019) fabricated via electrospinning, *Plantago major Mucilage* (PMM) blended with PVA, in order to produce an electrospun nanofiber. The researchers optimized the electrospinning parameters (voltage, tip-to-collector distance, feed rate, and PMM/PVA ratio) to obtain nanofibers with an average diameter of 250 nm. The viscosity, electrical conductivity, and surface tension of the PMM/PVA solution were 550 Cp, 575 μS/cm, and 47.044 mN/m, respectively [162]. The systems seem promising for biomedical applications. However, the researchers did not evaluate the effects of the fibers for CRC treatment.

#### 6.4.4. Polyzwitterions

Polyzwitterions are a class of polymers consisting of zwitterionic moieties (anionic and cationic groups) as monomers. Poly(sulfobetaines), poly(carbobetaines), and poly(phosphobetaines) are representatives of a special class of polyzwitterions [163]. Gromadzki et al. (2017) developed a core-shell nanocapsule consisting of a dimer fatty acid-based aliphatic polyester core and zwitterionic poly(sulfobetaine) shell for the controlled delivery of curcumin, obtaining nanoparticles of <100 nm in size with an encapsulation efficiency of 98%. The researchers evaluated free and encapsulated curcumin for cytotoxicity and antioxidant activity in a panel of human cell lines and rat liver microsomes, respectively. Encapsulated curcumin had superior cytotoxic and antioxidant activity versus the free drug. In addition, cell viability experiments with non-loaded nanoparticles, both coated and noncoated, demonstrated that the developed nanoparticles are nontoxic, making them potentially suitable candidates for systemic passive targeting in cancer therapy, namely for the treatment of solid tumors exhibiting a high tumor accumulation of the capsules due to enhanced permeability and retention effects [164].

#### 6.4.5. Polyoxazoline

Poly (2-oxazoline) (POZ) is a class of polymers formed by cationic ring-opening that were first identified and synthesized over 50 years ago. These polymers are nonionic, biostable, and water-soluble, and some are polar organic solvents. POZ can be synthesized from readily available non-toxic materials. The interest in using POZ in medical devices and drug delivery is very recent, and their application in multiple platforms is now being recognized by drug delivery scientists [165]. Although there is limited literature about the use of POZ for cancer treatment, some formulations for the treatment of skin cancer are reported. For instance, Simon et al. (2019) developed ointments with quercetin (core) encapsulated in POZ (shell). The POZ produced a stable formulation of spherical nanocapsules of 18 nm in size. Moreover, a good quercetin encapsulation (94% ± 4%) efficiency was observed with these nanosystems, allowing its homogeneous distribution in the nanocapsule.Therefore, Q-MM can be used as a reservoir of quercetin. Once loaded, quercetin’s impact on cancer cell viability was doubled while its antioxidant efficacy was preserved [166]. Accordingly, POZ are an alternative for the encapsulation of antioxidants; hopefully, some new advances in oral drug formulations will be presented in the following years that can be used for CRC.

#### 6.4.6. Polypeptides and Polyaminoacids

Polyaminoacids (PAA) and polypeptides (PPD) are polydisperse structures formed by the condensation of amino acid monomers through amide bonds that, contrary to proteins, cannot fold into globular or fibrillar structures [167]. In addition, they can carry versatile reactive functional groups on their side chains (carboxylic acid, hydroxyl, amino, and thiol groups) that allow for a variety of chemical modifications and compatibility with a wide range of bioactive compounds. Some outstanding nanocapsules of polyaminoacids have been developed for cancer treatment. For instance, Choi et al. (2018) encapsulated Celastrol (antioxidant) in PEGylated polyaminoacid-capped mesoporous silica nanoparticles for mitochondria-targeted delivery in solid tumors [107], and Patsula et al. (2019) modified maghemite nanoparticles with poly (l) poly(l-lysine) to protect the iron dioxide core from reaction with the encapsulated antioxidant (epigallocatechin-3-gallate from tea) and to promote the internalization of the nanoparticle of the system into the cancer cell [168].

PPD are special polymers that exhibit antioxidant properties themselves; in Table 3, some recent studies are presented.

Regarding proteins, there are reports of silk proteins that are able to stabilize polar and non-polar antioxidants, due to the amphiphilic properties of fibroin. For example, Lou et al. (2016) used fibroin to stabilize vitamin C, curcumin, and epigallocatechin gallate. The results indicated that these antioxidants presented improved environmental stabilities of up to 14 days due to the binding of antioxidant molecules to the hydrophobic or the hydrophilic/hydrophilic boundary regions of silk [174]. Despite this work not being directly focused on CRC, it is highly relevant because antioxidants can easily react with air and other environmental conditions, losing their ROS -scavenging properties, prior to be consumed by the patient; without stabilization, the antioxidant simply will have little or no effect in the patient. Lerdchai et al. (2016) designed a mixture of Thai silk fibroin/gelatin (denaturalized protein) sponges for the dual controlled release of curcumin and docosahexaenoic acid for localized cancer treatment. The sponges were fabricated by freeze-drying and glutaraldehyde cross-linking techniques. The highly cross-linked and slowly degrading silk fibroin/gelatin (50/50) sponge released curcumin and/or DHA at the slowest rate (for 24 days). Sponges were not toxic to L929 mouse fibroblasts, but a ratio of 1:4 (curcumin/docosahexaenoic acid) had the highest inhibitory effect on the growth of cancer cells [175]. Finally, Lozano-Pérez et al. (2017) encapsulated quercetin in silk fibroin nanoparticles (175 nm diameter). The nanoparticles had a negative surface charge that allowed the sustained release of the antioxidant that occurred throughout the experiment in both phosphate buffer saline (pH 7.4) and simulated intestinal fluid (pH 6.8) [176].

#### 6.4.7. Polyesters

Polyesters are polymers that contain the ester functional group in their main chain [156]. Polyesters are produced via condensation or ring-opening reactions. Due to the strong presence of oxygen groups, this polymer can poses a negative charge that can respond to pH changes [177]. Moreover, some polyesters can be enzymatically degraded such as in the case of polycaprolactone (PCL), polylactic acid (PLA), and polyhydroxyalkanoates (PHA), among others [178,179,180,181]. Lipases are an important group of esterases for the biodegradation of aliphatic polyesters. They are produced in the pancreas, liver, and digestive system to break down fat [180]. Fat and polyesters have the same functional groups (esters), which leads the enzyme to degrade these polymers. For CRC, antioxidants have been successfully encapsulated in PLA and in Poly (dl-lactic-co-glycolic) acid (PLGA). PCL and PHA have been employed via mixtures and copolymers, as described in Section 6.4.9.

Alippilakkotte et al. (2018) encapsulated curcumin in polylactic acid (PLA) using an eco-friendly emulsification-solvent evaporation strategy. The method resulted in an efficiency of around 90%. The in vitro release studies showed a sustained curcumin release, after a burst release, in the initial 12 h. The curcumin-loaded PLA nanocapsules could easily penetrate into the cancer cells and can cause a sustained drug release for effective cancer treatment [177].

Pereira et al. (2018) encapsulated Guabiroba phenolic extract in Poly (dl-lactic-co-glycolic) acid (PLGA) nanoparticles to improve the stability, bioavailability, and bioactivity of the extract. The encapsulated extract proved to present a higher antioxidant capacity compared to free extract. Moreover, a reduction in ROS generation in non-cancer cells was achieved with lower extract concentrations (*p* < 0.05) after encapsulation [181].

#### 6.4.8. Poly(vinylpyrrolidones)

Polyvinylpyrrolidone (PVP), commonly called polyvidone or povidone, is a water-soluble polymer made from the monomer *N*-vinylpyrrolidone by free-radical polymerization in the presence of AIBN as an initiator [182]. Dry PVP is a hygroscopic powder and readily absorbs up to 40% of water by its weight [182]. Nanofibers, particles, and films have been loaded with antioxidant extracts for drug delivery systems.

Sriyanti et al. (2017) and Andjani et al. (2017) developed electrospun nanofiber mats of polyvinyl(pyrrolidone) (PVP) with *Garcinia mangostana extract* (GME). The researchers found strong interactions of the PVP with the extract, which was molecularly dispersed in the electrospun PVP nanofiber matrix. The composite nanofiber mats exhibited very high antioxidant activities despite having been exposed to a high voltage during electrospinning [183,184]. A similar strategy was adopted by Andjani et al. (2017) and Zahra et al. (2019) using rotatory force spinning for encapsulating GME and Garlic (*Allium sativum*) extract; however, the obtained fibers were at the microscale [185,186].

Kamaruddin et al. (2018) developed sub-micron particles by the electrospraying of PVP and green tea extract. The researchers optimized the processes for obtaining the particles and saw the potential for drug delivery systems [186]. However, the particles were not tested for drug delivery applications for colorectal cancer. Similarly, Guamán-Balcázar et al. (2019) generated sub-micron particles of PVP with mango leaf extract, finding a relationship between the mango leaf extract/PVP ratio, temperature, and pressure of the supercritical antisolvent extraction process with the particle size (some of the particles were at the nanoscale). The in vitro desorption test showed a release profile of the extract components lasting up to 8 h under simulated intestinal fluids at pH 6.8 [187].

Contardi et al. (2019) produced a new material of PVP plasticized with p-coumaric acid for the encapsulation of bioactive compounds of interest in the pharmaceutical industry. An initial model of the encapsulation of carminic acid was evaluated, finding that by varying the ratio of PVP to p-coumaric acid, the release profile can be adjusted from minutes to hours; for instance, a ratio of 2:1 (PVP/p-coumaric acid) has a release profile lasting up to 70 h to obtain 100% release [188].

#### 6.4.9. Composites, Copolymers, and Mixtures

Composites and polymer mixtures take advantage of the synergystic effects of two polymers. For instance, one polymer may be highly compatible with the bioactive compound but not pH sensitive, thus the combination with another shell polymer will tailor the release profile. Some recent advances are presented below.

Thanyacharoen et al. (2018) developed a composite material of polyvinyl alcohol with chitosan to deliver gallic acid (antioxidant). The results seem promising as the gallic acid was released for periods longer than 16 h and its antioxidant properties were conserved [189].

Al-Ogaidi (2018) mixed two polysaccharides, alginate, and chitosan, to load vitamin C into nanoparticles of 25–30 nm in size. Moreover, Al-Ogaidi evaluated the effect of the pH on the overall size of the system, finding higher release at a pH of 6. Furthermore, it was found that entrapping vitamin C within the nanoparticle enhanced its anticancer activity [142]. Similar studies were carried out by Aluani et al. (2017) using quercetin instead of vitamin C [190]. Rahaiee et al. (2017) developed alginate–chitosan nanoparticles to stabilize crocin, an antioxidant with anticancer effects; crocin is highly sensitive to pH, heat, and oxidative stress, making its effectiveness reduced. The alginate–chitosan nanoparticles showed a controlled release profile in simulated gastrointestinal fluids and effectively protected crocin from the environment prior to being released [191].

Huang et al. (2016) improved the water dispersibility of curcumin, using core-shell nanoparticles of zein (core) and alginate–pectin (shell). The researcher found that curcumin-loaded core-shell nanoparticles were shown to have superior antioxidant and radical scavenging activities compared to curcumin solubilized in ethanol [192]. Likewise, Wei et al. (2019) developed zein–propylene glycol alginate–rhamnolipid composite nanoparticles to overcome the limitations of resveratrol such as water insolubility and chemical instability; the nanoparticles controlled the release for up to 2 h [193].

Arunkumar created nanocapsules of 100 nm using poly (lactic-co-glycolic acid)-polyethylene glycol to improve the solubility and stability of lutein (an antioxidant with poor solubility). The capsules showed higher stability and improved the bioability of the lutein, enhancing the antiproliferative effect of the antioxidant, evidenced by the lower lethal concentration (LC50) of 10.9 µM for the nanocapsules and 25 µM for free lutein [194].

Jaiswal et al. (2019) synthesized methyl methacrylate (MMA)-modified chitosan (CS) by a green method via a Michael addition reaction between CS and MMA in ethanol. The nanoparticles of approximately 100 nm had, in an in vitro drug release study, a maximal curcumin entrapment efficiency up to 68% with a high release at a pH of 5.0 and a lower one at physiological pH [195]. Positive charges on chitosan will generate maximum delivery at acid pH (stomach) rather than neutral (colon). Consequently, this strategy is not recommended for CRC treatment, since curcumin would be delivered before arriving at the desired area.

Eatemadi et al. (2016) developed a nanoparticle of PCL-PEG-PCL to encapsulate chrysin (antioxidant). The researcher investigated the effect of chrysin-loaded PCL-PEG-PCL on the T47D breast cancer cell line. The cell viability assay showed that chrysin has a time-dependent cytotoxic effect on the T47D cell line. Furthermore, the conducted studies showed that encapsulated chrysin has a higher antitumor effect on the gene expression of FTO, BRCA1, and hTERT than free chrysin [196]. This study was not focused on CRC, but these systems can be easily extrapolated to gastrointestinal drug delivery.

Wu et al. (2016) evaluated the structural, mechanical, antioxidant, and cytocompatibility properties of membranes prepared from PHA and arrowroot (*Maranta arundinacea*) starch powder (ASP). Furthermore, the researchers grafted acrylic acid to PHA (PHA-g-AA). The PHA-g-AA/ASP membranes had better mechanical properties than the PHA/ASP membrane. This effect was attributed to greater compatibility between the grafted PHA and ASP. The water-resistance of the PHA-g-AA/ASP membranes was greater than that of the PHA/ASP membranes, and a cytocompatibility evaluation with human foreskin fibroblasts indicated that both materials were nontoxic. Moreover, ASP enhanced the polyphenol content and antioxidant properties when they were encapsulated [197].

### 6.5. Polymers for Encapsulating Synthetics and Hybrid Adjuvants for CRC

Synthetics and hybrid adjuvants are based on 5-FU. As explained previously, 5-FU is the gold standard for cancer and CRC adjuvant treatment. However, along with the growing interest in natural antioxidants, new hybrid compounds derived from 5-FU conjugated with natural or synthetic molecules have been developed; for example curcumin, has been conjugated with 5-FU to act synergistically by enhancing cellular uptake and accumulation, by inducing the destabilization of the cytoskeleton and loss of mitochondrial membrane potential, initiating early and late apoptosis in cancer cells [198]. Furthermore, synthetic drugs can be mixed with other natural or synthetic compounds to potentiate them or reduce the side effects. An example of this strategy is the mixing of 5-FU with resveratrol to reduce the toxicity of 5-FU against healthy cells [199]. Examples of other hybrids and mixtures were reviewed by Carrillo et al. (2015); the paper can be consulted in the following reference: [200].

The first attempts at looking at polymer encapsulation techniques for 5-FU and its derivatives are limited, mainly due to 5-FU being first patented in 1957 and then the research into encapsulating the molecule being governed by pharmaceutical companies. However, today, most of the patents have expired, making these compounds attractive for developing drug delivery systems. According to Scopus, there are more than 2800 documents related to 5-FU drug delivery systems. In 2018, more than 180 papers were published, and among them, 114 include the word “polymer”. In the Table 4, some representative work from 2015 to 2019 is presented.

According to the above table, recent advances in synthetic adjuvants have been focused on improving the bioavailability of 5-FU. For example, for a pH-sensitive polymer that will deliver the 5-FU in the colonic area, the modification of the polymer surface with folic acid makes it selective for receptors that are more active in cancer cells, for targeted therapy. Moreover, the blending of polymers and bioactive compounds is a new approach to reducing the toxic side effects of 5-FU and enhancing its therapeutic effects.

## 7. Conclusions

Actual cancer treatment is helping to increase the survival rates and prognosis and, in some cases, to cure cancer in patients. Nevertheless, the fight against cancer is not over, especially for CRC, which is one of the most aggressive cancer types. Consequently, cancer is and will remain a hot topic in research.

Among the different adjuvant therapies for complementing or avoiding the surgical removal of the affected area, oral chemotherapy is the most convenient for patients and health professionals. Chemotherapy treatment is continuously evolving to reduce side effects and enhance the therapeutic effects of natural and synthetics drugs, using strategies involving the encapsulation of bioactive compounds. Furthermore, thanks to molecular biology and chemistry, researchers can quickly check the anticancer properties of bioactive compounds in vitro and in vivo in order to compare treatments.

Polymers are versatile biomaterials that can be loaded with CRC-targeting compounds and processed to tailor the desorption kinetics to respond to pH, enzymes, cellular receptors, and time, among others. Despite none of the studies aiming to compare response types (pH, enzymes, time, or cellular receptors), pH-responsive polymers are seemingly the most promising, so new research should be focused on studying polymer families with other ways of responding to stimuli. In vivo pharmacokinetics are a useful tool to compare polymers and bioactive compounds in order to optimize the therapeutic effects of such compounds.

Finally, it can be concluded that antioxidants are emerging compounds that, in the short term, will complement current chemotherapy treatments, and in the long term, these natural drugs will replace 5-FU and will play an important role in curing colorectal cancer.

## Figures and Tables

**Table 1:** Comparison of keyword groups in scientific papers for cancer adjuvants and colorectal cancer adjuvants. Two thousand papers were extracted from Scopus and examined in their keywords and the documents up to 31/12/2018; the keywords were grouped using VantagePoint.

Keyword Groups	Percentage	
	Cancer Adjuvants	Colorectal Cancer Adjuvants
Chemotherapy	41.2	45.3
Others adjuvant therapies	23.8	13.9
Drugs	10.4	28.0
Radiotherapy	19.9	9.2
Others	4.8	3.6

**Table 2:** Natural antioxidants for the prevention and treatment of colorectal cancer; recent reports from 2015 to 2019.

Natural Antioxidant	Authors	Main Findings	Reference
**Ginseng extracts**	Jin et al. (2016)	Meta-analysis indicated a significant 16% lower risk of developing cancer in patients who consumed ginseng.	[62]
	Wong et al. (2015)	Carcinogenic modulation. Cancer cells do not generate resistance to ginseng extracts. Kill cancer cells while exerting low toxicity toward healthy cells.	[63]
	Chong-Zin et al. (2016)	Ginseng extract enhanced the antiproliferative effect of 5-FU on human colorectal cancer cells.	[64]
	Tang et al. (2018) Kim et al. (2018)	Inhibits metastasis and reduces the invasion of CRC in vitro and in vivo.	[65,66]
**Ginger** **(Zerumbone)**	Sithara et al. (2018) Girisa et al. (2019)	Inhibits the proliferation of CRC cells (SW480) and thereby induced apoptosis, which might be due to mitochondria transmembrane dysfunction, translocation of phosphatidylserine, and chromatin condensation. Oral administration of zerumbone at more than 100 ppm for 17 weeks to mice significantly inhibited the multiplicity of and inflammation in colonic adenocarcinomas.	[67,68]
**Garlic**	Kim et al. (2019)	Diallyl disulfide from garlic increased tumor necrosis factor-related apoptosis in CRC cell lines and in vivo.	[69]
	Roy et al. (2018)	S-allyl-l-cysteine-sulfoxide (SACS)/alliin compounds from garlic showed higher affinity towards EGFR in silico, and in vitro, showed anticancer activity modulating the EGFR in CRC cells.	[70]
**Lactoferrin**	Li et al. (2017)	Lactoferrin inhibited cell viability, with the 50% concentration of inhibition at 81.3 ± 16.7 mg/mL and 101 ± 23.8 mg/mL for HT29 and HCT8 cells, respectively. Moreover, lactoferrin reduces the relative tumor volume in mouse models compared with negative control.	[71]
	Sugihara et al. (2017)	Rats given 500 and 1000 mg/kg/day of lactoferrin harbored significantly fewer colon aberrant crypt foci, adenomas, and adenocarcinomas than the rats from the control group, due to lactoferrin inhibiting cell growth and TNF-α mRNA expression.	[72]
**Polyphenols**	Yang et al. (2016)	Polyphenol (−)-epigallocatechin-3-gallate from green tea inhibited growth and the activation of the VEGF/VEGFR axis in human colorectal cancer cells.	[73]
	Gómez-Juaristi et al. (2017)	Yerba mate tea flavonoids are highly adsorbed and metabolized by the human body, especially in the colon microbiota.	[74]
	Amigo-Benavent et al. (2017)	Yerma mate, its phenolic components, and metabolites decrease cancer cell viability and proliferation; evaluated in vitro in Caco-2 colon cells.	[75]
	Schmit et al. (2016)	Coffee consumption was associated with 26% lower odds of developing colorectal cancer.	[76]
	Amigo-Benavent et al. (2017)	Green coffee bean, its phenolic components, and metabolites decrease cancer cell viability and proliferation; evaluated in vitro in Caco-2 colon cells.	[75]
	Scafuri et al. (2016)	Apple phenolic compounds interfere with the activity of nucleotide metabolism and methylation enzymes, similarly to some classes of anticancer drug.	[77]
	Darband et al. (2018)	The polyphenol quercetin poses anticancer effects in colon cancer; it inhibits cell proliferation, angiogenesis, and tumor metastasis, along with promoting apoptosis and autophagy and reducing the drug resistance.	[78]
	Huang et al. (2017)	Curcumin enhances the effects of irinotecan on CRC cells through the generation of reactive oxygen species and activation of the endoplasmic reticulum stress pathway.	[79]
	Ravindranathan et al. (2018)	Curcumin and oligomeric proanthocyanidins offer superior anti-tumorigenic properties in CRC, affecting DNA replication, the cell cycle, and mismatch repair in CRC cells.	[80]
	Marjaneh et al. (2018)	Combination of curcumin with 5-FU dramatically reduced the tumor number and tumor size in both the distal and middle parts of colon in colitis-associated CRC. Additionally, curcumin suppressed colonic inflammation and notably recovered the levels of antioxidant activity.	[81]
	Agudelo et al. (2017)	Polyphenols in *Vaccinium meridionale Swartz* juices showed an apoptotic effect on SW480. The caspase 3 activity was increased in a time-dependent manner in SW480-treated cells; the proapoptotic proteins were increased by 1.6- to 2.0-fold. In addition, SW480 cells significantly increased the production of intracellular ROS, parallel with a reduction in the intracellular content of glutathione (GSH) and consequently a decrease in the GSH/oxidized glutathione (GSSG) ratio.	[82]
	Buhrmann et al. (2018) Buhrmann et al. (2019)	Resveratrol suppressed the formation of cancer-like stem cells in two different CRC lines, and this was accompanied with a significant increase in apoptosis. Moreover, resveratrol suppresses the tumor necrosis factor B, which is a pro-carcinogenic compound.	[83,84]
**Fermented skim milk**	Chang et al. (2019)	*Lactobacillus paracasei subsp. paracasei* NTU 101-fermented skim milk in combination with chemotherapy for CRC in vivo significantly suppressed tumor growth and metastasis compared to chemotherapy alone via regulating vascular endothelial growth factor, matrix metalloprotein-9, and tissue inhibitor of matrix metalloproteinase-1 levels.	[85]
***Rosa canina* extracts**	Turan et al. (2018)	*Rosa canina* extract exhibited a selective cytotoxic effect on CRC cells compared with normal colon cells. The extract induced cell cycle arrest at the S phase and apoptosis via reducing matrix metalloproteinases in CRC cells.	[86]
**Vitamin C**	Aguilera et al. (2018)	Vitamin C uncouples the Warburg metabolic switch in KRAS mutant colorectal cancer, inducing apoptosis.	[87]
**Chlorophyll**	Semeraro et al. (2018)	Chlorophyll a is a good candidate for photodynamic therapy due to its intense absorption of red and near-infrared light. In combination with β-cyclodextrins, it was demonstrated that it selectively kills CRC via a necrotic mechanism.	[88]
**Piperin**	Bantal et al. (2018)	Piperin at 50 mg/kg reduced CRC’s effects in vivo (mouse model), i.e., inflammation and focal congestion in sub-mucosa and muscularis layers.	[89]
***Manilkara Zapota* extract**	Tan et al. (2019)	*Manilkara Zapota* extract leaf water extract can inhibit the viability of CRC cells in 72 h, at a concentration ranging from 21 to 84 µg/mL.	[90]

**Table 3:** Recent advances in polypeptides (PPD) with antioxidant effects for CRC.

Polypeptide	Authors	Main Findings	Reference
***Arca subcrenata* Polypeptides**	Hu et al. (2019)	*Arca subcrenata polypeptides* (PAS) inhibited the growth of HT-29 cells with an LC50 value of 117 µg/mL after 48h treatment, and significantly suppressed the tumor growth in nude mice bearing xenografted HT-29 cells at a dosage of 63mg/kg, with little influence on normal colon cells and normal colonic mucosa. PAS were then inspiringly found to induce apoptosis and G2/M phase arrest in HT-29 cells. The effect’s mechanism involved in the inhibition of IGF-1/IGF-1R signaling activation, which was responsible for inactivating the downstream Akt/mTOR pathway. PAS significantly inhibited the ATP production of HT-29 cells both in vitro and in vivo.	[169]
**Legume Seed Polypeptides**	Lima et al. (2016)	Albumin and globulin fractions from legume seeds were screened for MMP-9 inhibitors (enzymes related to cancer growth and metastasis). Lupin seeds contain the most efficient MMP-9 inhibitors of all legume seeds analyzed, inhibiting both gelatinases and HT29 migration and growth, while pea seeds showed no effect. Results reveal legume protein MMPIs as novel metalloproteinase inhibitors of possible pharmacological interest.	[170]
**Black Soybean Peptides**	Chen et al. (2019)	The peptide fractions that were collected in each step were tested for their antioxidant capacity and anticancer activities against cancer cell lines. The most active fraction with a molecular weight of 455.0 Da showed the highest free radical scavenging and hydroxyl radical scavenging activity with LC50 values of 0.12 and 0.037 µM, respectively. Moreover, it showed high cytotoxic potential against cancer cells. The amino acid sequence was identified as Leu/Ile-Val-Pro-Lys (L/I-VPK).	[171]
**Polypeptides from Intestinal Digestion of Germinated Soybean**	González-montoya et al. (2018)	The protein concentrate from germinated soybean was hydrolyzed with pepsin/pancreatin and fractionated by ultrafiltration. Whole digest and fractions > 10, 5–10, and < 5 kDa caused cytotoxicity to Caco-2, HT-29, and HCT-116 human colon cancer cells, and reduced inflammatory responses caused by lipopolysaccharide in macrophages RAW 264.7. Antiproliferative and anti-inflammatory effects were generally higher in 5–10 kDa fractions. The most potent fraction was mainly composed of β-conglycinin and glycinin fragments rich in glutamine.	[172]
**Sweet Potato Protein Hydrolysates**	Zhang et al. (2018)	Six sweet potato protein hydrolysates (SPPH) showed certain antiproliferative effects on HT-29 cells. Specifically, Alcalase exhibited the highest antiproliferative effect with the lowest LC50 value of 119.72 µg/mL. SPPH by Alcalase were further separated into four fractions (> 10, 5–10, 3–5 and < 3 kDa). Fractions < 3KDa showed the strongest antiproliferative effects, which were 43.87% at 100 µg/mL (*p* < 0.05). The <3 kDa fractions could cause G2/M cell cycle arrest with increased p21 expression and induce apoptosis via decreasing Bcl-2 expression, increasing Bax expression, and inducing caspase-3 activation in HT-29 cells. In addition, <3 kDa fractions could significantly inhibit the cell migration of HT-29 cells.	[173]

**Table 4:** Recent advances in using 5-fluorouracil (5-FU) and its derivatives in polymer encapsulation strategies for CRC.

Compounds	Polymers	Highlights	References
**5-FU Mixed with Doxorubicin (Dox)**	Dendritic nanomicelle of poly lactic acid (core) and polyamidoamine dendron (shell)	The nanocapsule has a diameter of 68.6 ± 3.3 nm and shows a pH-sensitive drug release behavior. The parallel activity of 5-FU and Dox shows synergistic anticancer efficacy.	[201]
**5-FU**	Core-shell nanocapsules; core: mesoporous silica; shell: chitosan/PEG	Drug loading (0.15–0.18 mg of 5FU/mg capsule). Controlled release profiles (15–65%) over 72 h. Cell specific cytotoxicity in cancer cells.	[202]
	Nanoparticles of PLGA conjugated with folic acid	Lower LC50 for encapsulated 5-FU against HT-29 cancer cells compared with free 5-FU. Folic acid on the surface of the nanoparticles induces a rapid intake of the nanoparticle into the cell.	[203]
	Complex of casein-coated iron oxide nanoparticles and folic acid-conjugated chitosan-graft-poly (2-dimethylaminoethyl methacrylate)	Lower toxicity to normal cells. pH-sensitive nanoparticles. Magnetic-sensitive nanoparticles.	[204,205]
	Loaded β-cyclodextrin-carrying polymeric poly(methylmethacrylate)-coated samarium ferrite nanoparticles		
	Zirconium metal organic nanoparticles (5-FU encapsulated in the crystal structure of Zirconium) coated with PEG	The encapsulation system is photosensitive, releasing the drug in response to the light	[206,207]
	5FU conjugated with chitosan		
	Non-coated and chitosan-coated alginate beads in a 3D printed tablet of polyacrilates	Controlled release of 5-FU from the alginate beads encapsulated within the hollow pH-sensitive tablet matrix at pH values corresponding to the colonic environment.	[208]
	Electrospum nanofibers of PCL/chitosan	High chitosan ratios led to increasing the drug release period. The release mechanism for all nanofibers was Fickian diffusion according to Korsmeyer–Peppas model.	[208]
	Crosslinked Sesbaniam gum (polyssacharide)	pH-responsive encapsulation system for colon-specific release.	[209,210,211,212,213]
	Carboxymethyl chitosan-grafted-poly (Acrylic Acid)-based		
	5-FU poly (l-lactide) composite by supercritical CO_2_ antisolvent		
	ZnO/carboxymethyl cellulose/chitosan nanocomposite beads		
	Azo hydrogels consisting in acryloyl chloride copolymerized with polyacrylates		
	Carboxylic curdlan and chitosan	Spherical morphology with an average size of about 180 nm and a zeta potential of around 41 mV. Encapsulation efficiency (86.47%) and loading content (10.81%).	[214]
**5-FU and Metformin (ME)**	Injectable hydrogels of PEG-b-poly(l-lysine)	In vitro degradation and drug release studies demonstrated that both ME and 5FU were released through hydrogels in a controlled and pH-dependent manner. The hydrogels had synergistic inhibitory effects on the cell cycle progression and cell proliferation in colon cancer cells, resulted from a combination of p53-mediated G1 arrest and apoptosis in C26 cells.	[215]
**5-fluorouracil and Oxaliplatin**	Poly (3-hydroxybutyrate-co-3-hydroxyvalerate acid)/poly (lactic-co-glycolic acid)	Higher anticancer activity using encapsulated drugs over free drugs. The nanoparticles are hemocompatible. Platform for co-delivery of anticancer compounds.	[210]
**Doxorubicin**	Lycium barbarum polysaccharides	The doxorubicin release from the nanoparticles was pH-dependent and was accelerated by decreasing pH. Cytotoxicity study showed that the loaded nanoparticles have significantly enhanced cytotoxicity in vitro, especially for human cancer cell lines.	[216]
**5-FU and Curcumin**	Chitosan/reduced graphene oxide nanocomposites	Higher encapsulationn efficiency (>90%). The synergistic cytotoxicity was observed upon addition of 5-FU and curcumin loaded in the nanocomposite, which shows the effectiveness of the system toward the inhibition of growth of HT-29 colon cancer cells. Better cytotoxicity with an LC50 of 23.8 μg/mL was observed for the dual-drug-loaded nanocomposite.	[217]
